# Recommendation for a Pilot MCDA Tool to Support the Value-Based Purchasing of Generic Medicines in the UAE

**DOI:** 10.3389/fphar.2021.680737

**Published:** 2021-06-08

**Authors:** Mohamed Naser Farghaly, Sara Ahmad Mohammad Al Dallal, Ahmad Nader Fasseeh, Nahed AbdulKhaleq Monsef, Eldaw Abdalla Mohamed Ali Suliman, Mohamed Attia Tahoun, Sherif Abaza, Zoltán Kaló

**Affiliations:** ^1^ Dubai Health Insurance Corporation, Dubai Health Authority, Dubai, United Arab Emirates; ^2^ Doctoral School of Sociology, Eötvös Loránd University, Budapest, Hungary; ^3^ Syreon Middle East, Alexandria, Egypt; ^4^ Strategy & Corporate Development Sector, Dubai, United Arab Emirates; ^5^ Health Regulation Sector, Dubai, United Arab Emirates; ^6^ Established Pharmaceuticals Division, Abbott Laboratories, Dubai, United Arab Emirates; ^7^ Syreon Middle East, Cairo, Egypt; ^8^ Center for Health Technology Assessment, Semmelweis University, Budapest, Hungary; ^9^ Syreon Research Institute, Budapest, Hungary

**Keywords:** pharmaceutical policy, off-patent pharmaceuticals, value-based health care, multi-criteria decision analysis, Dubai Health Authority, United Arab Emirates

## Abstract

**Introduction:** In recent periods the United Arab Emirates (UAE) has strengthened economic measures in its pharmaceutical policy by promoting local manufacturing and facilitating the use of generic medicines. International examples indicate the importance of quality control elements in the implementation of cost containment policies. Multicriteria Decision Analysis (MCDA) is increasingly used in health care to facilitate health care decision based on multiple objectives. Our objective was to develop a pilot MCDA tool for repeated use to support the value-based purchasing of generic medicines in the UAE.

**Methods:** An international evidence framework was adapted to UAE in a multistakeholder workshop organized by Dubai Health Authority. After validating the relevance of nine criteria in the local jurisdiction, participants decided the ranking and weight of each criterion by anonymous voting.

**Results:** The top four criteria focused on quality elements starting with real-world clinical or economic outcomes (with 19.8% weight), followed by the quality assurance of manufacturing (17.3%), then evidence on the equivalence with the original product (14.8%), and drug formulation and stability (12.3%). The pharmaceutical acquisition cost criteria ranked fifth with 9.4% weight. The bottom four criteria, including reliability of drug supply, macroeconomic benefit, pharmacovigilance and added value services related to the product had similar weights in the range of 5.5–7.7%.

**Conclusion:** Policy-makers in Dubai put high emphasis of value-based health care by incentivizing manufacturers of off-patent pharmaceuticals to generate additional scientific evidence compared to the mandatory minimum and acknowledging efforts to improve quality standards. The MCDA tool is considered suitable to improve the transparency and consistency of decision making in UAE for off-patent pharmaceuticals, and subsequently for other health technologies.

## Introduction

The UAE has a comprehensive, government-funded health service and a rapidly developing private health sector that delivers a high standard of health care to the population. Health care in the UAE is funded mainly by the government, however, private health care financing plays a crucial role in outpatient care especially in Dubai and Abu Dhabi (Koornneef et al., 2017).

In Dubai healthcare is regulated at both the federal level by the Ministry of Health and local level by the Dubai Health Authority (DHA) (Hamidi, 2015). DHA has an intention to facilitate value-based health care by fulfilling and promoting the regulatory’s vision and mission of transforming Dubai into a leading healthcare destination by enhancing consumer centricity, efficiency, innovation and accountability. The Insurance System for Advancing Healthcare in Dubai (ISAHD) initiative promotes two main pillars of ensuring the provision of health care relevant to population needs, while monitoring enhancements toward a sustainable high quality healthcare system (About ISAHD, 2020). Notably, ISAHD, in Arabic means “bringing happiness.” The monitoring system is based on the availability of high-quality electronic health care data (AlMarzooqi et al., 2020), which also provides an opportunity to generate real world evidence supported by artificial intelligence methods (Alhashmi et al., 2020).

In the United Arab Emirates (UAE), most pharmaceutical products are imported from around the globe under the regulatory framework of the Ministry of Health. In recent periods economic measures in the local pharmaceutical policies have been strengthened by promoting local manufacturing and facilitating the use of generic medicines (Balasubramanian et al., 2015).

External price referencing is applied to interpret the Emirati pharmaceutical prices in comparison with the prices in the country of origin, in Gulf and other countries (such as the United Kingdom) in the reference basket (Holtorf et al., 2019). The final UAE price should not exceed prices in other GCC member states, and a maximum price difference of 20% is allowed compared with other reference countries. However, in exceptional cases deviation from this general rule is possible.

Internal price referencing is applied for chemically and pharmacologically similar products registered in the UAE. Locally produced generic products must be priced 30% lower than the innovator or market leader medicine and imported generics must have at least a 40% price discount (Hasan and Lessing, 2015).

The efficiency of generic drug policies is usually assessed by measuring the level of price erosion and market share of generics (Kaló et al., 2015). Indeed, off-patent pharmaceuticals (OPPs) represent increasing proportion of the pharmaceutical market in many emerging countries. According to recent DHA data in the UAE the utilization of OPPs has also been increasing by 12% in value and by 10.2% in volume between 2015–2019. The volume growth rate of OPPs is above the 6.4% growth of the pharmaceutical market, consequently the market share of generic medicines was increased from 26.9% in 2015 to 31% in 2019 in standard units. The continuous increase in population size and extended insurance coverage to new expatriate workers ahead of Expo 2021 contributes to growth rate of OPPs. The increasing market share of OPPs in UAE are attributable to governmental policies and incentives to encourage local manufacturers on pharmaceutical manufacturing, implementation of International Nonproprietary Names (INN) prescribing and internal reference pricing of reimbursed medicines.

In 2019, over 61% of the UAE’s market share in standard units on prescribed OPPs were toward chronic disease; yet, OPPs had significantly higher market share in acute diseases, such as antiinfective and gastrointestinal medicines. On the other hand the utilization of OPPs is still relatively low in specialty areas with high growth rate, such as oncology (Radwan et al., 2018) or diabetes mellitus (Regmi et al., 2020). Increased utilization of generic medicines in the inpatient market segment is expected with the introduction of prospective payment (i.e., DRG system) in hospital financing in 2020.

Whilst cost-savings due to price erosion is an important benefit of utilizing generic medicines, drug quality, equivalence with the originator medicine, drug formulation, supply reliability, medical adherence and persistence, real world health outcomes and non-pharmaceutical costs also contribute to the success of generic drug policies (Kaló et al., 2015). Ignoring these variables can potentially result in inferior health outcomes and diminish the reduction in health care expenditure. Selection of preferred OPPs based on multiple criteria can facilitate value-based decision-making in this important market segment.

In 2015 a group of international experts selected 22 potential criteria that could be included into multicriteria decision analysis frameworks of OPPs (Brixner et al., 2017). By considering non-redundancy, non-overlap and preference independence, several criteria were removed or merged, leaving nine criteria in the international evidence framework for off-patent pharmaceutical review (EFOR) (Brixner et al., 2018). In the EFOR two criteria (equivalence with the reference medicine and product stability and expiry) are directly related to the product quality, and two criteria (pharmaceutical acquisition cost price, real world outcomes and costs) can help to judge the differential value of OPPs. Three criteria (manufacturing quality, supply reliability and contribution to the local economy) can be applied to evaluate the manufacturers’ overall quality, whereas two criteria (pharmacovigilance and value-added services) can be considered to assess services provided by manufacturers.

Our objective was to take a step forward in the implementation of value-based health care in UAE by developing a pilot MCDA tool for repeated use to support the value-based purchasing of OPPs (excluding biosimilar medicines) based on the international evidence framework.

## Methods

**FIGURE 1:**
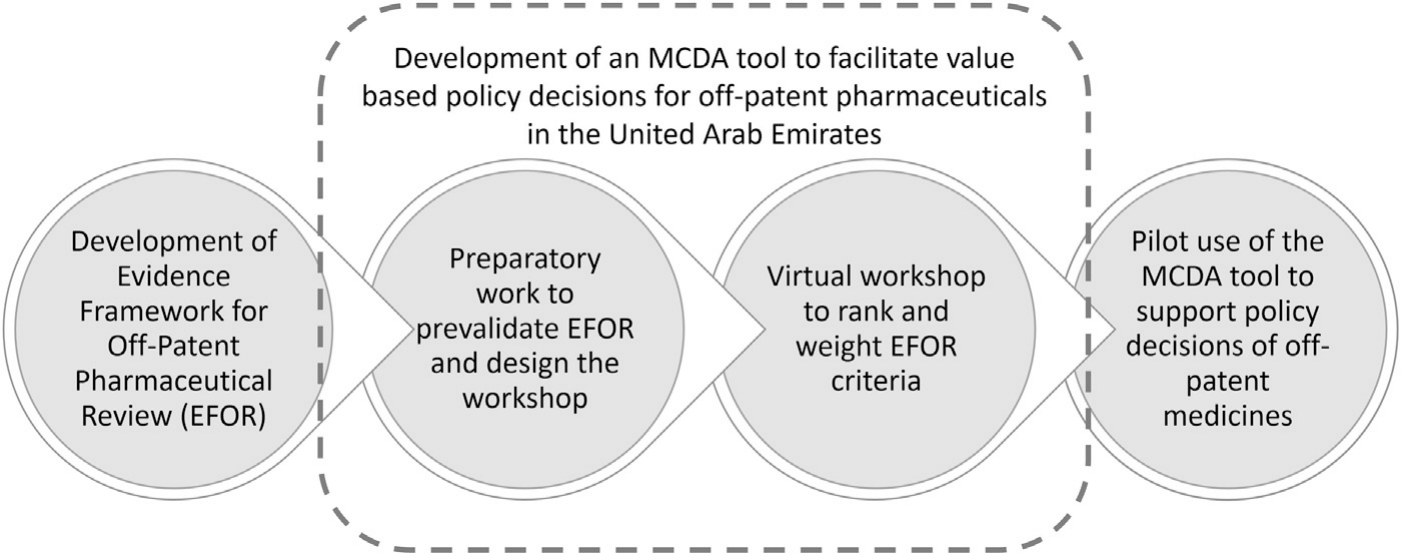
Working approach to develop a pilot MCDA tool to support policy decisions of off-patent pharmaceuticals in Dubai.

The process of developing the MCDA tool to facilitate value-based policy decisions for off-patent pharmaceuticals in Dubai is described in Figure 1.

### Preparatory Work

The preparatory work between leading pharmaceutical policy experts of Dubai Health Authority (DHA) and international experts contributing to the EFOR development started in January 2020. After concluding on the objectives of DHA, a two-day workshop structure was selected for the development of the MCDA tool by final selection, ranking and weighting of decision criteria. Due to the COVID pandemic, the face-to-face workshop had to be postponed and finally replaced by a one-day virtual workshop. To facilitate the workshop, a special Excel tool was prepared to lead workshop participants through each step.

### Virtual Workshop

A virtual workshop was hosted by the Dubai Health Authority on October 1, 2020. Local stakeholders were present at the meeting room of DHA by keeping appropriate social distancing. International MCDA experts—with responsibility of facilitating the workshop without any influence on voting—were connected by webstream.

The workshop started with an introductory session to introduce the concept of MCDA and the evidence framework for OPPs to the participants and an overview on pharmaceutical policies and market trends in UAE.

Preformed questions on the Mentimeter platform allowed the international moderator to implement the anonymous voting remotely. The voting results were presented after all participants submitted their vote to avoid consensus bias (Ross et al., 1977). After each voting step average values were entered in the Excel based MCDA tool to facilitate the interpretation of results and ensure transparency.

As a first step participants were asked to provide consent their anonymous answers could be aggregated and used in the scientific publication.

The first voting question explored the number of criteria included in the MCDA tool. After that, participants voted for the threshold of price differential compared to the cheapest OPP alternative.

The next step was weight elicitation in two phases, in which participants first established an order of importance of the criteria by applying the SMART method. After selecting and ranking the most important three criteria, the second most important three criteria were selected and ranked and finally the bottom three criteria were ranked. The final round of voting explored the relative incremental weight of each criterion compared to the previous criterion (i.e., swing weighting), then the final weights were calculated by normalizing the sum of weights to 100% (Németh et al., 2019).

The Excel tool included a test case which allowed participants to understand how the MCDA tool works in practice, to test its reliability and to discuss whether any amendments should be considered.

## Results

Out of the 21 participants, 19 decision-makers represented the public sector and the remaining two participants represented the private insurance sector. The majority of participants were mainly representing Dubai’s regulatory and policy makers from the Dubai Health Insurance Corporation, the Dubai’s strategy and corporate development sector and Health Regulation Sector. All participants gave consent about publishing the aggregated anonymous answers.

At first, participants voted about the number of criteria on the MCDA tool. 57% of the participants voted for not excluding any of the nine proposed criteria, while 19% participants voted for the inclusion of eight criteria, 24% participants voted for the inclusion of only seven criteria. Therefore, with majority voting all nine EFOR criteria were included to the MCDA tool.

Subsequently, participants voted for 204% threshold of price differential compared to the cheapest OPP alternative. This means that if the price of the cheapest alternative is 100 AED, a product with 202 AED price would receive 50% of scores for the price criterion, and any medicines above 304 AED price is not eligible for any scores of the price criterion.

Finally, participants completed the SMART ranking and swing weighting exercise. Results of the criteria ranking, and weighting are presented in Table 1. The top four criteria focused on quality elements starting with real-world clinical or economic outcomes (with 19.8% weight), followed by the quality assurance of manufacturing (17.3%), then evidence on the equivalence with the original product (14.8%), and drug formulation and stability (12.3%). The pharmaceutical acquisition cost criteria ranked fifth with 9.4% weight. The bottom four criteria, including reliability of drug supply, macroeconomic benefit, pharmacovigilance and added value services related to the product, had similar weights in the range of 5.5–7.7%. Pharmacovigilance and macroeconomic benefit were ranked the same and consequently received the same weights.

**TABLE 1:** Ranking and weight of criteria in the pilot MCDA tool to facilitate policy decisions of off-patent pharmaceuticals in Dubai.

Criteria	Ranking	Incremental weight to the previous criterion	Final weight (%)
Real-world clinical or economic outcomes	1	14.5%	19.8
Quality assurance of manufacturing	2	16.8%	17.3
Equivalence with the references (original) product	3	20.5%	14.8
Stability and drug formulation	4	30.3%	12.3
Pharmaceutical acquisition cost (price)	5	23.2%	9.4
Drug supply reliability	6	16.1%	7.7
Macroeconomic benefit (local investment)	7	0.0%	6.6
Pharmacovigilance	8	19.5%	6.6
Added value service related to the product	9	—	5.5

## Discussion

MCDA is increasingly used in health care globally to improve the consistency and transparency of policy decisions (Thokala et al., 2016). MCDA has been applied in some pilot studies in the Middle East (Ghandour et al., 2015; Al-Badriyeh et al., 2016; Fouad and El Mourdy, 2017; Bakhtiari et al., 2020; Öztürk et al.,), in the future broader utilization of this methodology is expected in the region (Fasseeh et al., 2020).

The intention of DHA to facilitate value-based health care is reflected in the choices of the MCDA tool for OPPs. The four most highly rated criteria focus solely on quality elements starting with real-world clinical or economic outcomes, followed by the quality assurance, then the equivalence with the reference product and closing with stability and drug formulation, leaving no more quality elements in the MCDA tool. These criteria would incentivize manufacturers to generate additional scientific evidence for example from observational studies or database analyses compared to the mandatory minimum and acknowledging efforts to improve quality standards. In fact, it is unlikely that many pharmaceutical companies can submit clinical trial or real-world evidence about the incremental benefits or improved stability or drug formulating of their OPPs. Such products can be considered value-added medicines, hence DHA intends to take a pioneer step to acknowledge evolutionary innovation of generic medicines (Petykó et al., 2020).

Surprisingly, the pharmaceutical price criterion ranked only fifth with 9.4% weight. In Kuwait the price criterion in the MCDA tool had 35% weight, and in Indonesia 40% weight (Inotai et al., 2018; Abdullah et al., 2019). As opposed to these countries, all criteria in the DHA MCDA tool have more than 5% weight, which provides incentives to pharmaceutical companies by making improvement in each criterion.

It should be highlighted that the number of workshop participants was relatively small and may not be fully representative to all stakeholder groups with important contributions to pharmaceutical policies in Dubai. Hence, the MCDA tool for OPPs can be considered as a starting point for utilizing MCDA in healthcare decision-making in the UAE, which should be tested in pilot settings. The real-world experience during the pilot phase could then provide a basis for necessary adjustments of the MCDA tool in another workshop with a preferably broader representation of different stakeholder groups, including eminent pharmacists and physicians. Subsequently the MCDA tool can be used routinely in pharmaceutical purchasing decisions. An if the tool proves to be useful to facilitate value-based purchasing of generic medicines, further MCDA tools may be developed with similar methodology to support the purchasing decisions of other health technologies, e.g., biosimilar medicines, orphan drugs, vaccines or medical devices.

## Data Availability

The original contributions presented in the study are included in the article, further inquiries can be directed to the corresponding author.
